# Automatic IMRT treatment planning through fluence prediction and plan fine-tuning for nasopharyngeal carcinoma

**DOI:** 10.1186/s13014-024-02401-0

**Published:** 2024-03-20

**Authors:** Wenwen Cai, Shouliang Ding, Huali Li, Xuanru Zhou, Wen Dou, Linghong Zhou, Ting Song, Yongbao Li

**Affiliations:** 1 School of Biomedical Engineering, Southern Medical University, Guangzhou, 510515 China; 2 Department of Radiation Oncology, Sun Yat-sen University Cancer Center, State Key Laboratory of Oncology in South China, Collaborative Innovation Center for Cancer Medicine, Guangdong Key Laboratory of Nasopharyngeal Carcinoma Diagnosis and Therapy, Guangzhou, 510060 China; 3 Zhujiang Hospital, Southern Medical University, Guangzhou, 510282 China

**Keywords:** Automatic planning, Nasopharyngeal carcinoma, IMRT, Fluence prediction, Plan fine-tuning

## Abstract

**Background:**

At present, the implementation of intensity-modulated radiation therapy (IMRT) treatment planning for geometrically complex nasopharyngeal carcinoma (NPC) through manual trial-and-error fashion presents challenges to the improvement of planning efficiency and the obtaining of high-consistency plan quality. This paper aims to propose an automatic IMRT plan generation method through fluence prediction and further plan fine-tuning for patients with NPC and evaluates the planning efficiency and plan quality.

**Methods:**

A total of 38 patients with NPC treated with nine-beam IMRT were enrolled in this study and automatically re-planned with the proposed method. A trained deep learning model was employed to generate static field fluence maps for each patient with 3D computed tomography images and structure contours as input. Automatic IMRT treatment planning was achieved by using its generated dose with slight tightening for further plan fine-tuning. Lastly, the plan quality was compared between automatic plans and clinical plans.

**Results:**

The average time for automatic plan generation was less than 4 min, including fluence maps prediction with a python script and automated plan tuning with a C# script. Compared with clinical plans, automatic plans showed better conformity and homogeneity for planning target volumes (PTVs) except for the conformity of PTV-1. Meanwhile, the dosimetric metrics for most organs at risk (OARs) were ameliorated in the automatic plan, especially D_max_ of the brainstem and spinal cord, and D_mean_ of the left and right parotid glands significantly decreased (*P* < 0.05).

**Conclusion:**

We have successfully implemented an automatic IMRT plan generation method for patients with NPC. This method shows high planning efficiency and comparable or superior plan quality than clinical plans. The qualitative results before and after the plan fine-tuning indicates that further optimization using dose objectives generated by predicted fluence maps is crucial to obtain high-quality automatic plans.

**Supplementary Information:**

The online version contains supplementary material available at 10.1186/s13014-024-02401-0.

## Background

Nasopharyngeal carcinoma (NPC) is one of the most common head and neck malignant tumors in East and Southeast Asia, and radiation therapy is the primary treatment modality for non-metastatic NPC because of its high sensitivity to ionizing radiation [1]. Intensity-modulated radiation therapy (IMRT) can accurately deliver radiation dose to targets while sparing adjacent normal organs with intensity modulation of high-energy photon beams so that it has favorable treatment outcomes for NPC [2, 3]. Nevertheless, serious complications frequently occur during or after IMRT, such as xerostomia [4], radiation caries [5], dysphagia [6], taste impairment [7], and radiation-induced brain injury [8]. Thus, balancing the high-dose coverage to targets and minimum-dose exposure to organs at risk (OARs) is crucial. However, the completion of IMRT treatment planning for geometrically complex NPC involving multiple OARs and non-convex planning target volumes (PTVs) is extremely challenging [9]. In clinical practice, IMRT treatment planning is a time-consuming inverse planning process completed in a treatment planning system (TPS) with manual trial-and-error fashion [10]. As a result, the quality of plan is largely influenced by the planner’s experience and skills, which implies that patients may receive diverse quality of treatment. Therefore, many studies on automatic treatment planning have been conducted to enhance plan quality consistency and improve planning efficiency for IMRT [11–14].

Knowledge-based planning (KBP) is an automatic planning method that has been integrated into commercial TPS to accomplish dose volume histogram (DVH) estimation using the built-in KBP model, and dose objectives are generated to guide the follow-up optimization process [14–17]. However, DVH prediction can only provide the relative volume received doses of certain structures without dosimetric spatial information, which would result in inferior plan dose distribution and dose conformity [18, 19]. This issue was further solved by predicting the 3D dose distribution from anatomical information of structures based on deep convolutional neural networks (CNNs), which showed fairly similar dosimetric quality to those in deliverable plans [13, 20–23]. However, the predicted dose distribution cannot be easily converted into voxel-level optimization objectives in current commercial TPS to generate the corresponding deliverable plan. Recent advances bypassed inverse optimization and directly predicted fluence maps to generate multi-leaf collimator (MLC) leaf sequence to obtain the final plan [24–28].

Although the KBP method based on fluence prediction can directly generate plans in TPS without inverse optimization, there is no guarantee that the resulting plan is optimal because any fluence prediction error, fluence loss during leaf motion calculation, and patient heterogeneity would result in plan quality degradation. In this study, we combined CNN-based fluence map prediction with script-based plan fine-tuning to automatically generate IMRT treatment plans for 38 patients with NPC. The plans were first generated by predicted fluence maps, and then further fine-tuned with dose objectives provided from the predicted fluence generated dose. Finally, we evaluated both the plan quality and planning efficiency for the proposed automatic planning method.

## Methods

### Patient collection

The ethics committee of Sun Yat-sen University Cancer Center approved the retrospective use of clinical treatment plans for patients in this study. A cohort of 38 patients with NPC treated with IMRT at Sun Yat-sen University Cancer Center between March 2015 and February 2016 was collected. Among these 38 patients, 30 (79%) were males and 8 (21%) were females, with an age range of 22–79 years (median age of 49 years). All IMRT plans were generated in the same treatment machine of Varian Trilogy system (Varian Medical Systems, Palo Alto, CA, USA) with Millennium 120 MLC, using nine equally spaced beams (beam angles at 0°, 40°, 80°, 120°, 160°, 200°, 240°, 280°, and 320°) and 6 MV photon beam energy in flattening filter mode.

All patients with NPC had multiple radiation targets, and five PTVs named “PTV-GTV,” “PTV-1,” “PTV-2,” “PTV-LN(L)” (PTV of left lymphonodus), and “PTV-LN(R)” (PTV of right lymphonodus) were considered. The prescription doses for PTV-GTV, PTV-1, PTV-2, PTV-LN(L), and PTV-LN(R) were 70, 60 or 64, 54 or 58, 60–70, and 60–70 Gy, respectively, in 30–33 fractions. Seventeen OARs used in this study were body, brainstem, spinal cord, chiasm, tongue, left and right optic nerves, left and right lens, left and right temporal lobes, left and right mandibles, left and right temporomandibular joints, and left and right parotid glands.

### Fluence prediction

A customized CNN model named “shared encoder network” proposed in our previous study was used for fluence prediction [29]. The shared encoder network constructed by one encoding path and two decoding paths was exploited to simultaneously generate dose distribution and fluence maps with structure contours and CT images as input. The contour of PTV was converted to a 3D mask according to the prescription dose, and the maximum prescription dose of PTVs where the voxel belonged was set to each voxel of the PTV mask and every non-PTV voxel was assigned zero. Each OAR was expressed as a binary mask with one set inside the contour and zero set outside the contour. We extracted CT image, PTV mask, and 17 OAR masks from each patient as input data, and we utilized the trained model to generate fluence maps with resolution of 2.5 mm × 2.5 mm and size of 160 × 160 at nine beam directions. The predicted fluence maps were saved in a file storage format with header information and pixel values before importing into TPS.

### Automatic plan generation

**Fig. 1:**
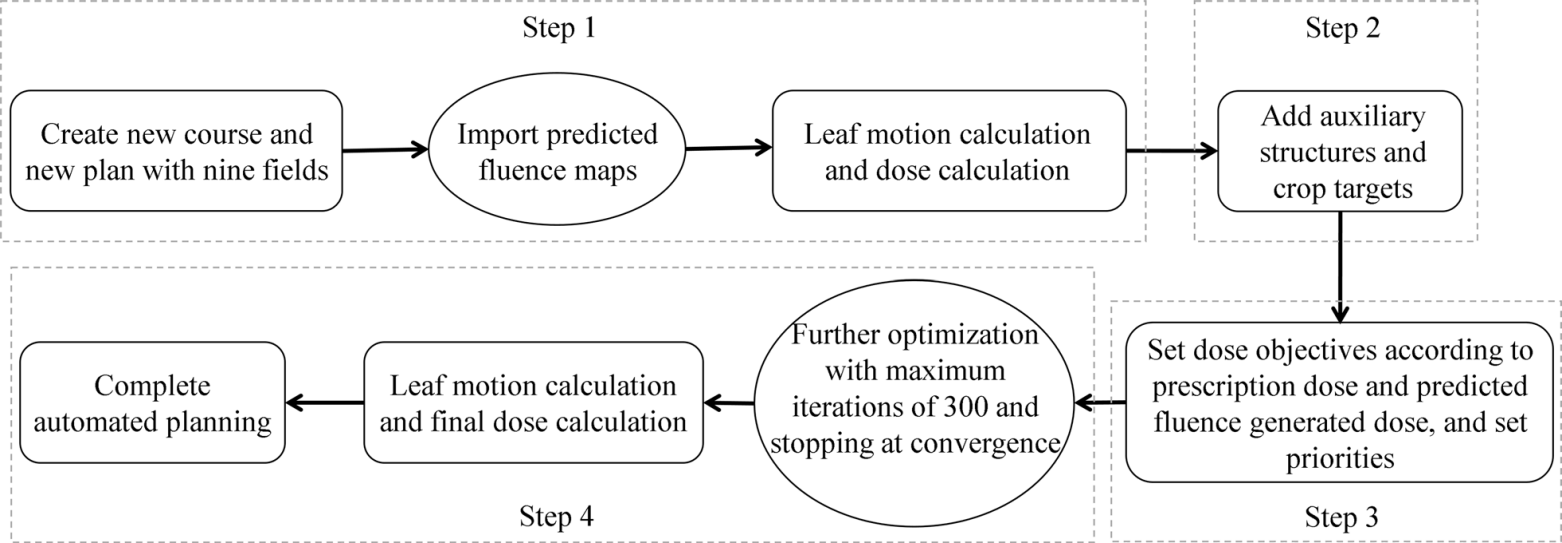
The flowchart of automatic plan generation

The automatic planning process was accomplished in a research-only Eclipse TPS (version 15.6). Using the Eclipse Scripting Application Programming Interface script to assist radiotherapy planning and plan quality assessment [30–32], we integrated all manual planning operations into a compiled C#-based script to achieve a fully automated planning process. With the customized C#-based script, the predicted fluence maps were imported into Eclipse to generate an initial plan, the auxiliary target structures were produced and dose objectives and priorities were set according to prescription and predicted fluence generated dose, the optimization and leaf motion and final dose calculations were also completed automatically. An approved binary plugin can be executed with one click to automatically generate a plan in the Eclipse system. Figure 1 demonstrates the procedure of an automatic IMRT plan generation.

*Step 1*: Importing predicted fluence and calculating dose distribution

After creating a new course and new plan for a selected patient, the predicted fluence maps for each beam were imported into Eclipse and then converted to MLC sequences with MLC leaf motion calculations (Varian LMC 15.6.03). The predicted fluence generated plan was obtained after calculating the resulted dose distribution with Anisotropic Analytic algorithm (AAA 15.6.03).

*Step 2*: Adding auxiliary structures and cropping targets

To improve target dose conformity and reduce the radiation dose to normal tissues, we added four auxiliary structures in optimization: “PTV-1-Crop,” “PTV-2-Crop,” “Ring 2 cm,” and “40 Gy-PTV2”. “PTV-1-Crop” was defined as the region of 3 mm outward expansion of PTV-GTV subtracted from PTV-1. “PTV-2-Crop” was generated by subtracting the 3 mm outward expansion of PTV-1, PTV-LN (L), and PTV-LN (R) from the whole region of PTV-2. “Ring 2 cm” was defined as a 2 cm-wide ring between PTV-2 expanded by 0.2 cm and PTV-2 expanded by 2.2 cm, and “40 Gy-PTV2” referred to the region between the isodose line of 40 Gy and the 0.3 cm extension of PTV-2. The Additional file 1 illustrates the definition of four auxiliary structures.

*Step 3*: Setting optimization objectives and priorities

The plan generated from predicted fluence maps already provided the achieved dose information, but the plan quality may need to be further improved. To ensure a plan quality improvement after plan fine-tuning, we set stringent optimization objectives (Table 1). The dosimetric values for key OARs were set 5%–25% lower than the achieved values from the predicted fluence generated plan.

**Table 1:** Optimization objectives were set according to prescription dose and predicted fluence generated dose information

ROI name	Objective type	Objective dose (Gy)	Priority
PTV-GTV	Maximum D_0%_	73.5	70
	Minimum D_100%_	71.5	150
PTV-1-Crop	Maximum D_0%_	PTV-1’ Px-dose + 3	70
PTV-1	Minimum D_100%_	PTV-1’ Px-dose	150
PTV-2-Crop	Maximum D_0%_	PTV-2’ Px-dose + 6	70
PTV-2	Minimum D_100%_	PTV-2’ Px-dose	150
PTV-LN(L)	Maximum D_0%_	PTV-LN(L)’ Px-dose + 3	70
	Minimum D_100%_	PTV-LN(L)’ Px-dose + 1.5	150
PTV-LN(R)	Maximum D_0%_	PTV-LN(R)’ Px-dose + 3	70
	Minimum D_100%_	PTV-LN(R)’ Px-dose + 1.5	150
Brainstem	Maximum gEUD, a = 20	Predicted gEUD × 0.85	50
Brainstem 3 mm	Maximum gEUD, a = 20	Predicted gEUD × 0.85	50
Spinal cord	Maximum gEUD, a = 20	Predicted gEUD × 0.85	50
Spinal cord 3 mm	Maximum gEUD, a = 20	Predicted gEUD × 0.85	50
Left normal parotid	Maximum gEUD, a = 3	Predicted gEUD × 0.75	50
Right normal parotid	Maximum gEUD, a = 3	Predicted gEUD × 0.75	50
Left optic nerve	Maximum gEUD, a = 1	Predicted gEUD	50
Right optic nerve	Maximum gEUD, a = 1	Predicted gEUD	50
Chiasm	Maximum gEUD, a = 1	Predicted gEUD	50
Ring 2 cm	Maximum D_1%_	Predicted dose × 0.95	50
	Maximum D_20%_	Predicted dose × 0.95	50
	Maximum D_50%_	Predicted dose × 0.95	50
	Maximum gEUD, a = 15	Predicted gEUD × 0.95	50
40 Gy-PTV2	Maximum D_1%_	Predicted dose × 0.95	50
	Maximum D_20%_	Predicted dose × 0.95	50
	Maximum D_50%_	Predicted dose × 0.95	50
	Maximum gEUD, a = 10	Predicted gEUD × 0.95	50

*Px-dose* prescription dose, *gEUD* generalized equivalent uniform dose

*Step 4*: Further optimization and calculating final dose distribution

Plan optimization was completed with the Photon Optimizer algorithm (PO, version 15.6.03) with continued optimization, and the dose distribution calculated from the predicted fluence was set as the intermediate dose to reduce the optimization convergence time. Plan optimization was completed with the maximum number of 300 iterations. After optimization, the optimal fluence maps were converted to MLC leaf sequences with MLC leaf motion calculations, and the final dose distribution was calculated to generate the final deliverable plan.

## Evaluation

The plan quality was quantitatively assessed between clinical plans, automatic plans with warm start (using predicted fluence as initial value for further optimization) and cold start (optimization with no initial state) for all 38 patients. Dosimetric metrics, including D_2%_, D_98%_, conformity index (CI) [33], and homogeneity index (HI) [34], were reported for five PTVs. The CI is expressed as CI = $\frac{{TV}_{RI}}{TV}$, where ${TV}_{RI}$ refers to target volume covered by the prescription dose, and *TV* is the target volume. The range of CI values is from 0 to 1, and high CI values indicate good target conformity. The HI is defined as $\frac{{D}_{5\%} - {D}_{95\%}}{{D}_{px}}$, where ${D}_{5\%}$ and ${D}_{95\%}$ are 5% and 95% of the PTV volume received dose, respectively, and ${D}_{px}$ is the prescription dose. In general, low HI values represent a homogeneous dose distribution inside the PTV. Maximum dose (D_max_) and mean dose (D_mean_) were used to assess quantitative metrics for 17 OARs. All dosimetric comparisons were tested for statistical differences using the Wilcoxon signed-rank test with a significance level of 0.05.

## Results

**Fig. 2:**
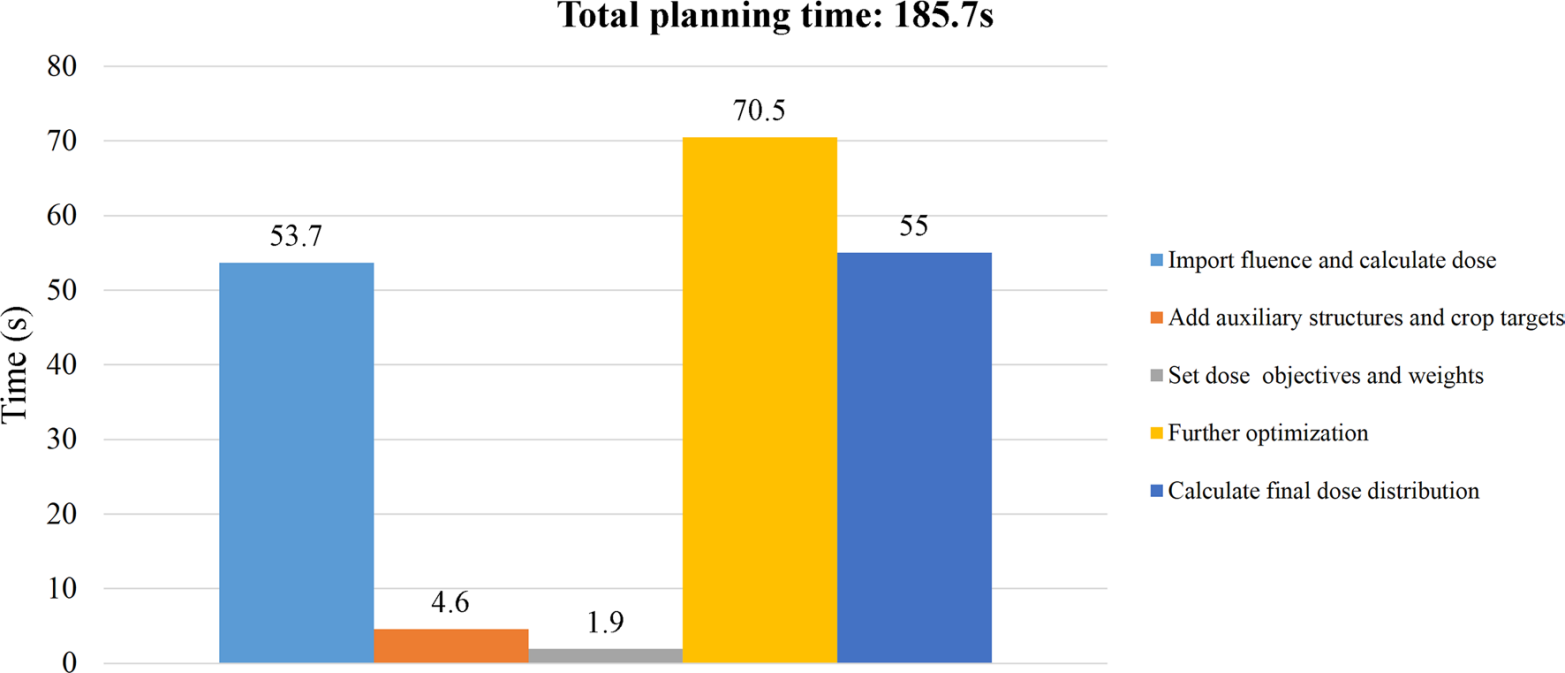
The time breakdown of automatic planning process for a randomly selected patient

The nine-field fluence maps predicted from the trained model took approximately 12 s for one patient. On average, the whole process of automatic planning in Eclipse using script per patient was completed in 199.8 s. Plan fine-tuning step with warm start didn’t show significant iteration number reduction and optimization efficiency improvement than cold start. The time cost of automatic planning for 38 patients ranged from 155.9 to 239.3 s, and the median time was 206.1 s. Figure 2 shows the time spent in each step of the automatic planning process for a randomly selected patient, and the total planning time was 185.7 s.

**Fig. 3:**
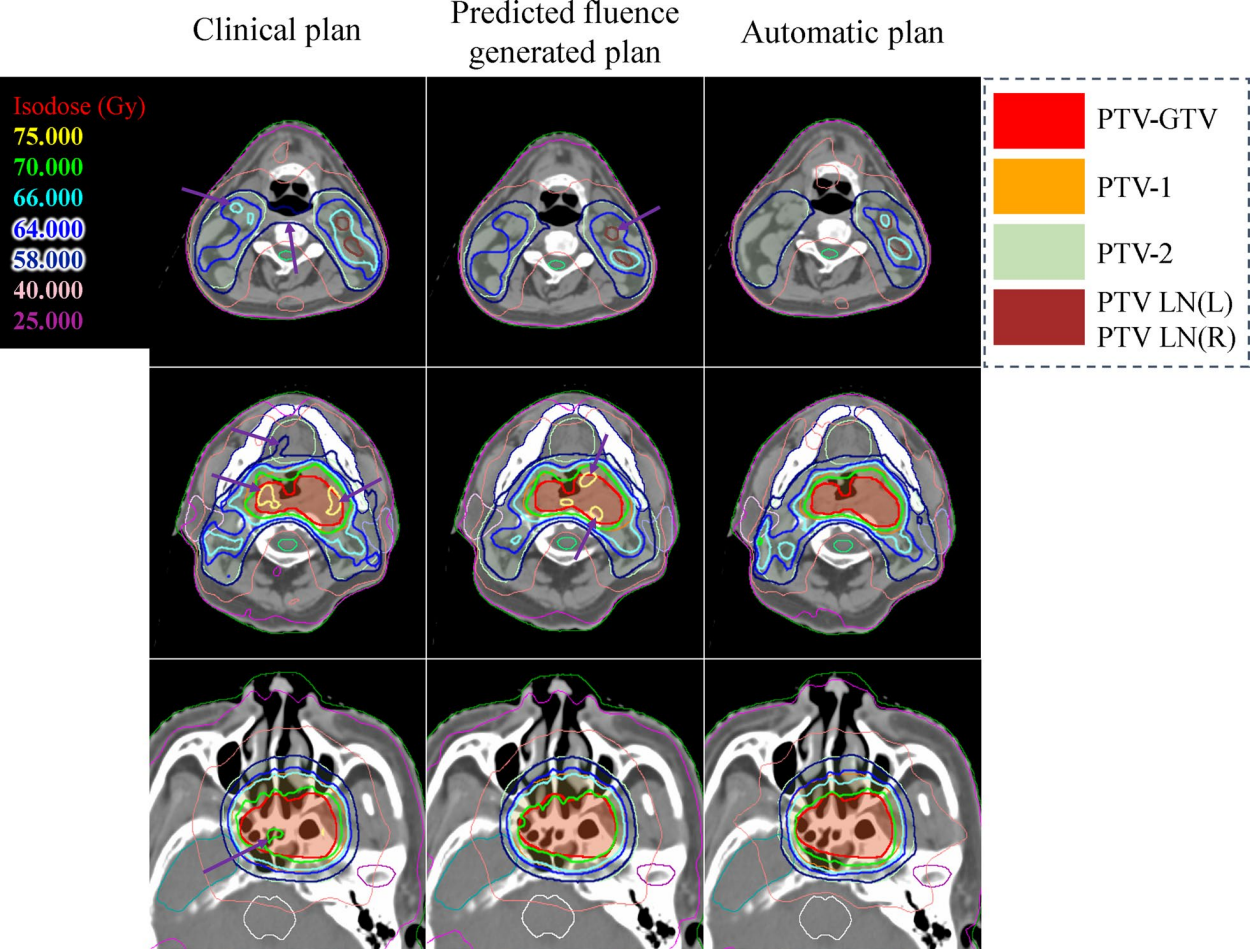
The comparison of dose distributions between clinical plan, predicted fluence generated plan and automatic plan for patient A. The first column is clinical result, the second column is predicted fluence generated result and the third column is automatic fine-tuning result

**Fig. 4:**
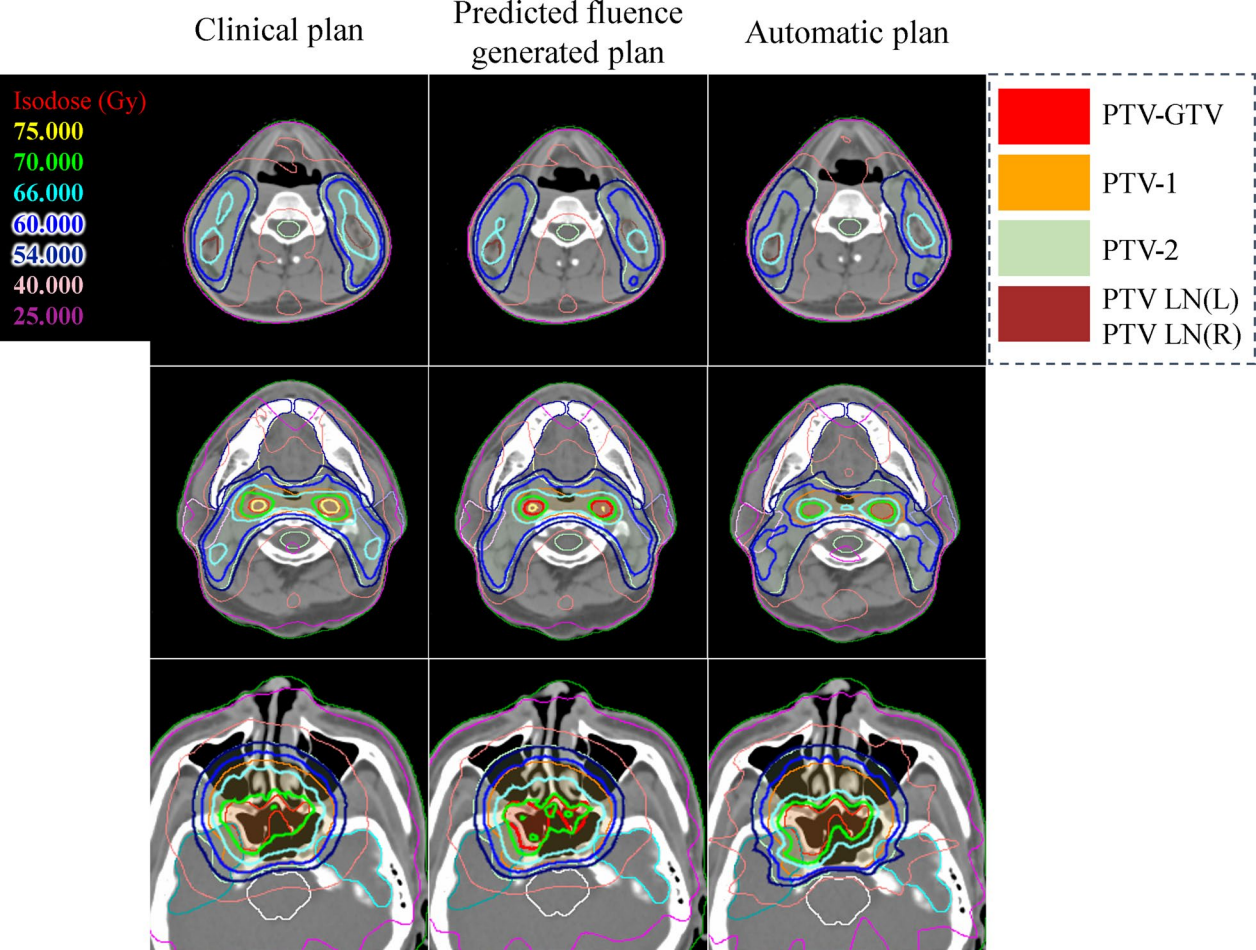
The comparison of dose distributions between clinical plan, predicted fluence generated plan and automatic plan for patient B. The first column is clinical result, the second column is predicted fluence generated result and the third column is automatic fine-tuning result

The dose distribution comparison among clinical plan, predicted fluence generated plan, and automatic plan for two representative patients (patient A and patient B) on three axial sections is illustrated in Figs. 3 and 4, respectively. In general, all three plans achieved comparable dose coverage on both PTV-GTV (red segments) and PTV-1 (orange segments), but the automatic plan further improved the target dose homogeneity and conformity as indicated by the arrows compared with the clinic plan and predicted fluence generated plan.

**Fig. 5:**
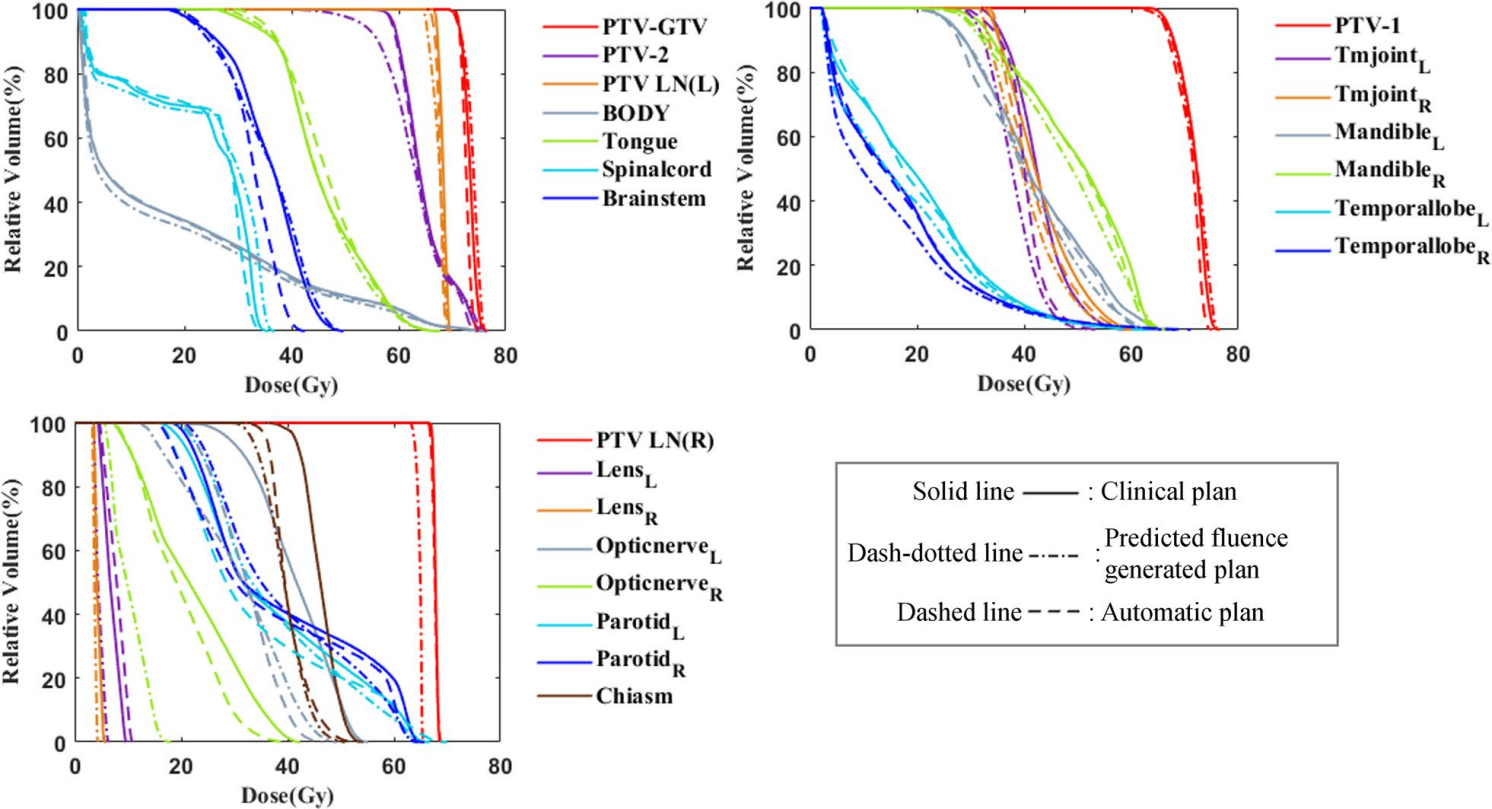
The comparison of DVH curves between clinical plan (solid line), predicted fluence generated plan (dash-dotted line) and automatic plan (dashed line) for patient A

**Fig. 6:**
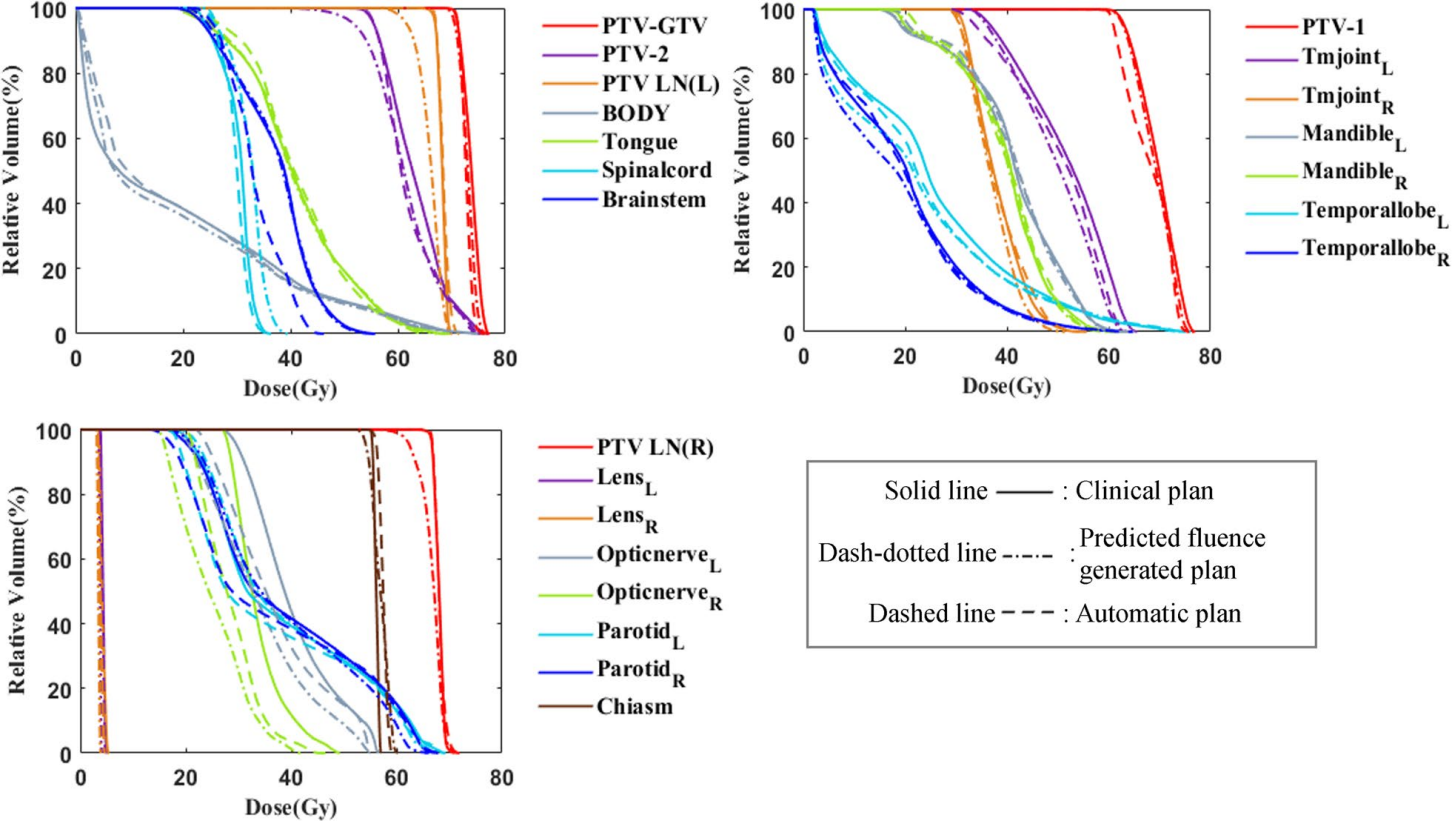
The comparison of DVH curves between clinical plan (solid line), predicted fluence generated plan (dash-dotted line) and automatic plan (dashed line) for patient B

Figures 5 and 6 show the DVH comparison of five PTVs and  seventeen OARs for the two patients, respectively. No significant difference was found in the target curves between the clinical plan (solid line) and automatic plan (dashed line). The predicted fluence generated plan (dash-dotted line) showed an obviously inadequate dose coverage for PTV-2, PTV-LN(L), and PTV-LN(R), whereas the automatic plan successfully recovered the target dose coverage after plan fine-tuning. For OARs, both the predicted fluence generated plan and automatic plan showed better dose sparing than the clinical plan.

**Fig. 7:**
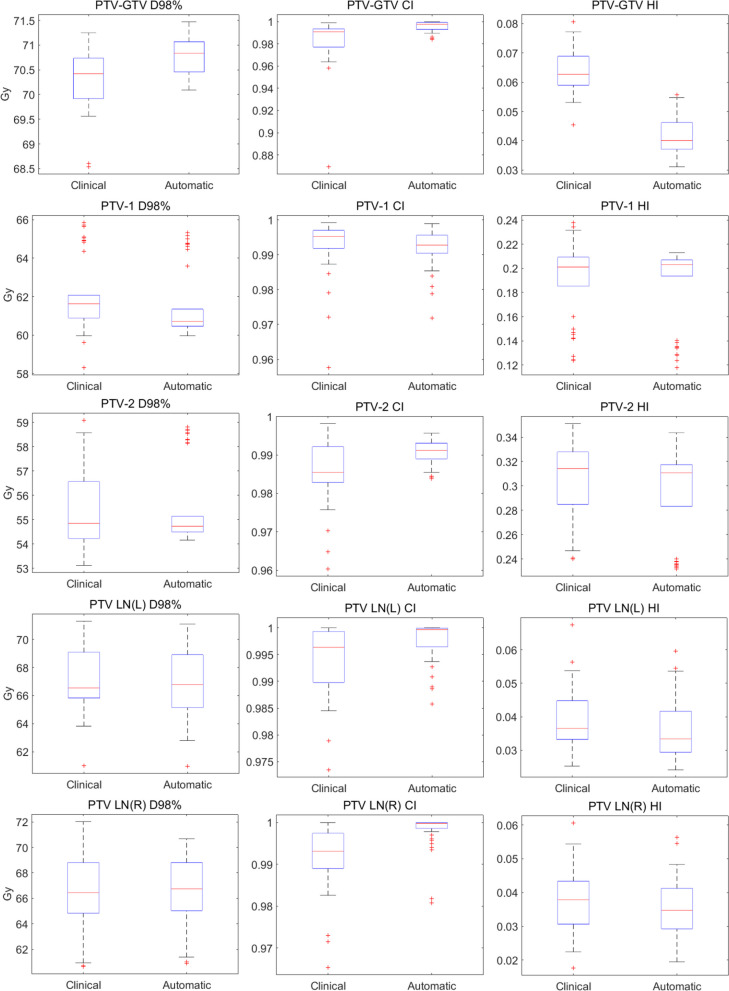
The box plot comparisons of D_98%_, CI and HI between clinical and automatic plans for five targets\

**Fig. 8:**
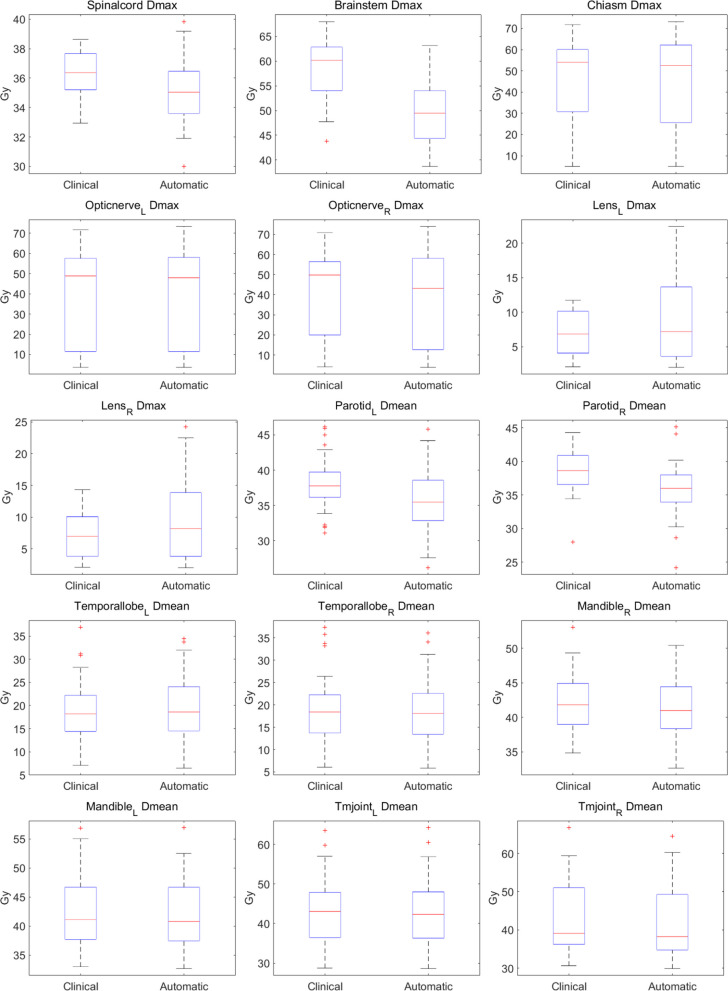
The box plot comparisons of dosimetric results between clinical and automatic plans for fifteen OARs

Figures 7 and 8 showed the comparison of major dosimetric results between clinical plans and automatic plans using box plots for 38 patients. Compared to automatic plans, the dosimetric parameters for the five targets in clinical plans generated using conventional planning methods exhibited a relatively more dispersed distribution range and worse plan quality consistency. In addition, automatic plans produced better target dose with lower D_2%_, higher D_98%_, higher CI, and lower HI except for D_98%_ and CI of PTV-1. For most OARs, automatic plans also showed lower dosimetric values than clinical plans, especially D_max_ of brainstem, spinal cord, left and right optic nerves, and chiasm and D_mean_ of left and right parotid glands.

Table 2 summarizes the comparison results of dosimetric metrics and corresponding p-values between clinical plans, automatic plans with warm start and cold start. The automatic plans with cold start also ameliorated the dosimetric results for most structures compared to clinical plans, and showed only a slight plan quality difference compared with automatic plan with warm start. However, automatic plans with warm start showed higher plan MUs than automatic plans with cold start.

**Table 2:** The comparison of dosimetric metrics for thirty-eight patients in the unit of Gy (mean ± standard deviation) between clinical plans, automatic plans with warm start and cold start

Structures	Metrics (Gy)	Clinical plans	Automatic plans with warm start	Automatic plans with cold start	P1	P2	P3
PTV-GTV	D_2%_	75.80 ± 0.56	74.54 ± 0.53	74.38 ± 0.71	** < 0.001**	** < 0.001**	0.62
	D_98%_	70.30 ± 0.58	70.79 ± 0.36	69.31 ± 0.82	** < 0.001**	** < 0.001**	**0.002**
	CI	0.983 ± 0.02	0.996 ± 0.00	0.939 ± 0.05	** < 0.001**	** < 0.001**	** < 0.001**
	HI	0.064 ± 0.01	0.042 ± 0.01	0.058 ± 0.01	** < 0.001**	0.32	** < 0.001**
PTV-1	D_2%_	75.43 ± 0.57	74.14 ± 0.52	74.06 ± 0.07	** < 0.001**	** < 0.001**	0.92
	D_98%_	62.10 ± 1.88	61.57 ± 1.81	61.06 ± 2.32	** < 0.001**	** < 0.001**	** < 0.001**
	CI	0.993 ± 0.01	0.992 ± 0.01	0.963 ± 0.02	0.06	** < 0.001**	**0.004**
	HI	0.191 ± 0.03	0.187 ± 0.03	0.188 ± 0.04	0.08	0.16	0.54
PTV-2	D_2%_	74.47 ± 0.70	73.38 ± 0.53	73.42 ± 0.68	** < 0.001**	**0.002**	0.56
	D_98%_	55.52 ± 1.76	55.54 ± 1.67	55.58 ± 1.91	0.54	0.06	0.31
	CI	0.986 ± 0.01	0.991 ± 0.00	0.979 ± 0.01	**0.001**	**0.002**	** < 0.001**
	HI	0.304 ± 0.03	0.295 ± 0.03	0.292 ± 0.03	** < 0.001**	0.16	**0.01**
PTV-LN(L)	D_2%_	70.31 ± 2.87	70.15 ± 2.83	70.72 ± 2.76	0.32	**0.03**	**0.03**
	D_98%_	67.10 ± 2.42	67.13 ± 2.46	68.10 ± 2.09	0.56	**0.004**	**0.006**
	CI	0.994 ± 0.01	0.998 ± 0.00	0.999 ± 0.00	** < 0.001**	** < 0.001**	0.16
	HI	0.039 ± 0.01	0.036 ± 0.01	0.033 ± 0.01	**0.04**	0.06	0.08
PTV-LN(R)	D_2%_	69.72 ± 3.06	69.56 ± 2.73	70.32 ± 2.78	0.59	** < 0.001**	** < 0.001**
	D_98%_	66.58 ± 2.81	66.71 ± 2.70	67.92 ± 2.84	**0.03**	** < 0.001**	**0.007**
	CI	0.992 ± 0.01	0.998 ± 0.00	0.999 ± 0.00	** < 0.001**	** < 0.001**	0.69
	HI	0.038 ± 0.01	0.035 ± 0.01	0.030 ± 0.01	0.20	**0.006**	0.08
Brainstem	D_max_	58.51 ± 5.89	50.17 ± 6.43	51.29 ± 6.25	** < 0.001**	** < 0.001**	** < 0.001**
Spinal cord	D_max_	36.28 ± 1.46	35.21 ± 2.33	35.15 ± 2.71	**0.002**	0.08	0.43
Chiasm	D_max_	47.01 ± 19.41	45.42 ± 22.04	46.60 ± 21.11	0.26	0.71	0.47
Left optic nerve	D_max_	39.99 ± 22.17	39.69 ± 23.64	40.57 ± 22.44	0.68	0.28	0.09
Right optic nerve	D_max_	40.36 ± 21.96	38.83 ± 23.20	39.79 ± 22.07	0.07	0.77	0.12
Left len	D_max_	6.83 ± 3.04	8.53 ± 5.26	10.24 ± 5.5.82	**0.001**	** < 0.001**	**0.01**
Right len	D_max_	7.12 ± 3.54	9.32 ± 6.32	11.15 ± 7.17	** < 0.001**	** < 0.001**	**0.01**
Left parotid gland	D_mean_	38.24 ± 3.87	35.73 ± 5.03	36.16 ± 5.20	** < 0.001**	**0.01**	**0.002**
	D_median_	33.03 ± 5.87	30.51 ± 7.91	30.54 ± 7.43	**0.002**	** < 0.001**	0.61
Right parotid gland	D_mean_	38.38 ± 3.11	35.83 ± 4.00	37.49 ± 4.51	** < 0.001**	0.06	** < 0.001**
	D_median_	32.81 ± 4.26	29.85 ± 5.84	31.04 ± 6.49	** < 0.001**	0.13	** < 0.001**
Left temporal lobe	D_mean_	18.80 ± 6.96	19.00 ± 7.47	19.34 ± 8.0	0.98	**0.02**	**0.004**
	D_median_	15.08 ± 7.73	15.32 ± 8.21	16.01 ± 9.04	0.67	**0.004**	**0.006**
Right temporal lobe	D_mean_	19.23 ± 7.49	18.80 ± 7.38	19.84 ± 7.64	**0.01**	0.19	** < 0.001**
	D_median_	15.85 ± 8.77	15.19 ± 8.02	16.26 ± 8.97	**0.04**	0.43	0.004
Left mandible	D_mean_	42.74 ± 6.00	42.18 ± 5.85	43.27 ± 5.35	**0.004**	0.23	** < 0.001**
	D_median_	43.24 ± 6.44	42.50 ± 6.54	43.93 ± 6.70	**0.008**	**0.04**	** < 0.001**
Right mandible	D_mean_	41.87 ± 4.45	41.17 ± 4.40	42.30 ± 4.10	** < 0.001**	0.08	** < 0.001**
	D_median_	42.33 ± 4.72	41.46 ± 4.74	42.99 ± 4.53	** < 0.001**	**0.04**	** < 0.001**
Body	D_mean_	18.73 ± 4.11	18.65 ± 4.22	18.44 ± 3.74	0.24	**0.002**	0.09
	D_median_	9.29 ± 6.13	9.84 ± 6.29	8.04 ± 5.01	** < 0.001**	** < 0.001**	** < 0.001**
Tongue	D_mean_	43.00 ± 4.32	43.67 ± 4.24	42.74 ± 4.74	**0.001**	0.63	0.06
	D_median_	42.27 ± 3.82	43.28 ± 3.92	41.82 ± 4.64	** < 0.001**	0.77	**0.01**
Left temporo-mandibular joint	D_mean_	43.34 ± 8.59	43.03 ± 8.42	41.84 ± 8.48	0.07	** < 0.001**	**0.02**
	D_median_	42.73 ± 9.07	42.72 ± 8.87	40.85 ± 7.31	0.46	0.06	** < 0.001**
Right temporo-mandibular joint	D_mean_	42.50 ± 9.15	41.25 ± 8.88	41.93 ± 10.15	** < 0.001**	0.11	0.42
	D_median_	42.13 ± 9.69	40.79 ± 9.03	41.58 ± 4.64	** < 0.001**	0.77	0.13
Plan	MU	1623 ± 302	1815 ± 206	1683 ± 146	** < 0.001**	0.56	**0.004**

*P1* significant difference between clinical and automatic plans with warm start, *P2* significant difference between clinical plans and automatic plans with cold start, *P3* significant difference of automatic plans between warm start and cold start. Results with *P* < 0.05 indicated statistical significance and were labeled with bold

## Discussion

The ideal trade-off between target coverage and OAR sparing for NPC is challenging and often requires a well-experienced planner to iteratively adjust optimization parameters during manual IMRT planning. Such a conventional method is time/resource-consuming and leads to uneven plan quality. In this study, we developed an automated IMRT plan-generating framework through fluence prediction and further plan fine-tuning, and we integrated it into commercial TPS via scripts to achieve automatic plan generation by one click. The proposed method was validated through 38 patients with NPC, showing high planning efficiency in less than 4 min and comparable plan quality with clinical plans.

Several previous studies have proposed to automatically generate plans based on direct fluence prediction [24–28], which may lead to unstable plan quality due to inaccurate prediction of fluence or quality loss when converting fluence into MLC sequences. The proposed plan fine-tuning step may be favored to further improve the plan quality. The DVH results in Figures 5 and 6 illustrated that some of the targets showed low-dose coverage in the predicted fluence generated plan, whereas the dose coverage significantly improved after the automatic plan fine-tuning step. Compared with the DVH prediction-based KBP method, the proposed method generated an initial deliverable plan first, which provided already achieved dosimetric information although may not optimal, while the predicted DVH is not always guaranteed to be achievable and optimal (uncertainties from machine learning models).

For NPC patients, VMAT is increasingly used in current clinical practice. Although the proposed method was only validated on IMRT plans in this study, it can be potentially used for VMAT plan optimization. Specifically, fluence can be predicted at discrete beam angles (such as 60 beams with 6 degree space) first, a VMAT plan arc sequencing step can be followed to generate an initial plan, then the plan fine-tuning step can be proceeded by using the predicted dose as objectives and the initial plan as warm start to generate a final plan. The planning efficiency improvement can be expected and would be more meaningful than IMRT. In the future study, we plan to extend the proposed method to automatic VMAT planning for NPC patients.

## Conclusions

In conclusion, we proposed an automated IMRT plan-generating method for patients with NPC through fluence prediction and further plan fine-tuning. This method remarkably reduced the dose for most OARs without compromising target conformity and homogeneity. Compared with clinical plans, the automatic plans showed high planning efficiency and achieved comparable or superior plan quality.

### Supplementary Information

**Additional file 1.** Supplementary figure and table.

## Data Availability

The datasets used during the current study are available from the corresponding author on reasonable request.

## Abbreviations

NPC: Nasopharyngeal carcinoma
IMRT: Intensity-modulated radiation therapy
PTV: Planning target volume
OAR: Organ at risk
TPS: Treatment planning system
KBP: Knowledge-based planning
DVH: Dose volume histogram
CNN: Convolutional neural network
MLC: Multi-leaf collimator
CI: Conformity index
HI: Homogeneity index
