# Supplementary material for: Automatic IMRT treatment planning through fluence prediction and plan fine-tuning for nasopharyngeal carcinoma
Source: Radiat Oncol. 2024 Mar 20;19:39. doi: 10.1186/s13014-024-02401-0 (PMC10956235; doi:10.1186/s13014-024-02401-0)
Supplement: Supplementary file 1 — Additional file 1. Supplementary figure and table. [file 13014_2024_2401_MOESM1_ESM.docx]

**Supplementary Material**

| 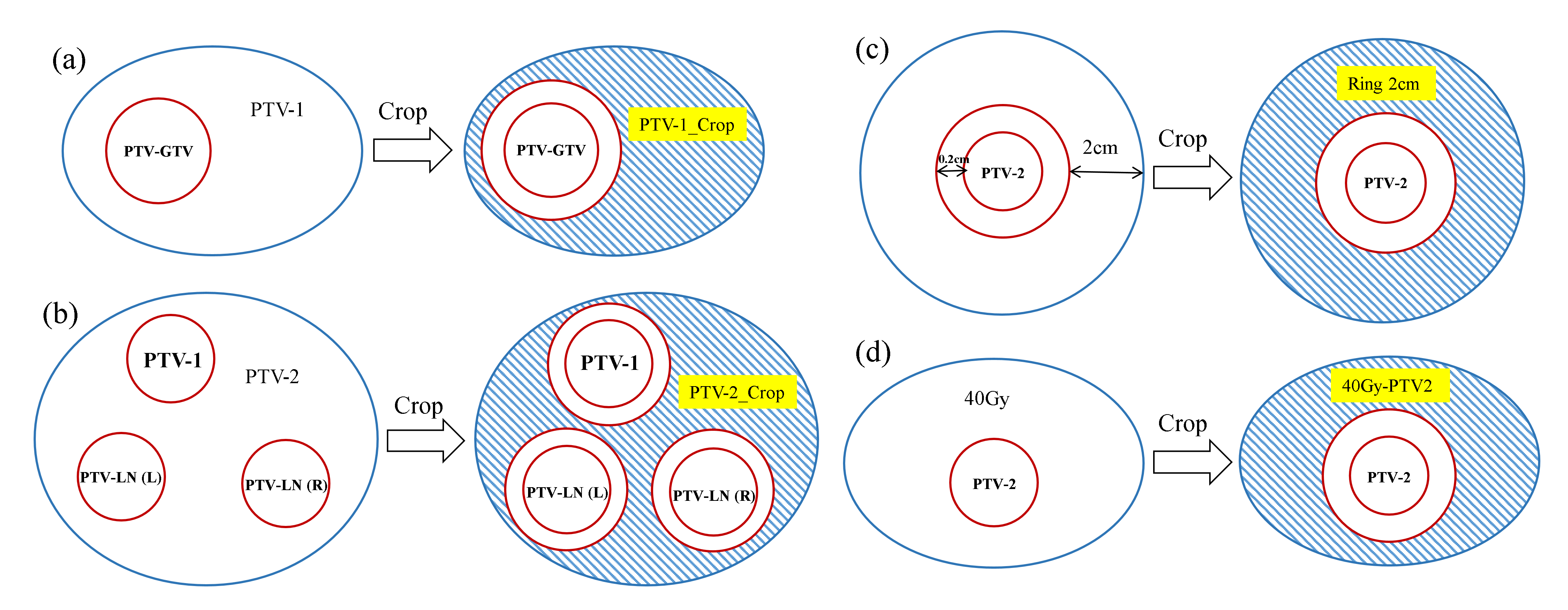  **Fig.** Definition of “PTV-1-Crop” (a), “PTV-2-Crop” (b), “Ring 2cm” (c) and “40Gy-PTV2” (d) respectively. |
| --- |
